# Evaluating the Risk Factors for *Porcine Epidemic Diarrhea Virus* Infection in an Endemic Area of Vietnam

**DOI:** 10.3389/fvets.2020.00433

**Published:** 2020-07-29

**Authors:** Thi Ngan Mai, Thanh Phong Bui, Thi My Le Huynh, Yosuke Sasaki, Shuya Mitoma, Hala El Daous, Watcharapong Fahkrajang, Junzo Norimine, Satoshi Sekiguchi

**Affiliations:** ^1^Graduate School of Medicine and Veterinary Medicine, University of Miyazaki, Miyazaki, Japan; ^2^Faculty of Veterinary Medicine, Vietnam National University of Agriculture, Hanoi, Vietnam; ^3^Branch of Cargill Vietnam Co., Ltd, Dong Van II Industrial Zone, Ha Nam, Vietnam; ^4^Department of Animal and Grassland Sciences, Faculty of Agriculture, University of Miyazaki, Miyazaki, Japan; ^5^Center for Animal Disease Control, University of Miyazaki, Miyazaki, Japan; ^6^Faculty of Veterinary Medicine, Benha University, Toukh, Egypt; ^7^Department of Livestock Development, Ministry of Agriculture and Cooperatives, Bangkok, Thailand; ^8^Department of Veterinary Science, Faculty of Agriculture, University of Miyazaki, Miyazaki, Japan

**Keywords:** *Porcine epidemic diarrhea virus*, case–control study, risk factor, endemic, Vietnam

## Abstract

*Porcine epidemic diarrhea virus* (PEDV) causes enteritis, vomiting, watery diarrhea, and high mortality in suckling pigs, threatening the swine industry. Porcine epidemic diarrhea (PED) re-emerged globally in 2013 in many important swine-producing countries in Asia and the Americas. Several studies have identified the risk factors for the spread of PEDV in acute outbreaks. However, limited information is available on the risk factors for the transmission of PEDV in endemic regions. We hypothesized that poor biosecurity, location, and some social or cultural practices are the main risk factors for PEDV transmission in the Vietnamese pig population. The aim of this study was to evaluate the potential risk factors for the transmission of PEDV in an endemic area in Vietnam. In this case–control study, questionnaires containing 51 questions were completed for 92 PEDV-positive and 95 PEDV-negative farms. A logistic regression analysis was performed to assess the risk factors associated with PEDV infection. Province and the total number of pigs were included as random effects to determine their influence on the risk of PEDV infection. Twenty-nine variables of interest that have been associated with PEDV status were analyzed in a univariate analysis (*P* <0.20), with backward stepwise selection. Only three of these 29 variables in four models remained significant PEDV risk factors in the final model: farrow-to-wean production type, distance from the farm to the slaughterhouse (<1,000 m), and the presence of chickens on site (*P* <0.05). This is the first study to identify the main risk factors for PEDV infection in an endemic area. Our findings suggest that hygiene measures should be strictly implemented on farms for the effective control and prevention of PEDV infection.

## Introduction

*Porcine epidemic diarrhea virus* (PEDV) causes enteritis, vomiting, watery diarrhea, and high mortality in <10-day-old suckling pigs (almost 100%) and significant economic losses in the swine industry (1). PEDV re-emerged in 2013 and caused huge economic losses globally, in many important swine-producing countries in North America (USA, Canada, and Mexico) (2–4) and Asia (South Korea, Japan, and Taiwan) (3, 5, 6). Direct contact between the pigs within farms is the primary transmission route of PEDV, via the fecal–oral route (7). Improvements in farm hygiene management and avoiding risky practices associated with contact with pig excrement are key factors in preventing the transmission of PEDV to farms (6). Several studies have comprehensively evaluated the risk factors for the spread of PEDV in the early phase of PEDV outbreaks (6, 8, 9). Indirect PEDV transmission through contaminated personal protection equipment occurs rapidly, within 24 h (10). In Japan, large-scale farms, proximity to an infected farm, number of feed trucks, short disinfectant contact time, and visiting veterinarians are factors strongly associated with the PEDV status of farms (11, 12). In the USA, transport is considered the main route of PEDV transmission (13, 14). In Italy, trucks have been shown to play an important role in the spread of PEDV (15). PEDV-contaminated feed was reported to be a significant risk factor for the transmission of PEDV between farms in the USA, Japan, and Canada (8, 9, 11, 16–18). Its transmission by transport vehicles was also reported to be a biological factor causing the rapid spread of PEDV in the USA and Japan (11, 13). Because PEDV is highly infectious and the infectious dose is low, it can be locally transmitted from PEDV-infected farms to neighboring PEDV-free farms through aerosol transmission or contaminated fomites (12, 19, 20). However, no study has analyzed the risk factors for PEDV spread in endemic areas or countries.

In Vietnam, PEDV was first observed in the southern provinces in 2009 (21). Published studies have demonstrated that PEDV is present in all major swine-producing regions in Vietnam (21–26). A descriptive survey recently provided evidence that northern Vietnam is an endemic area for PEDV, with a high proportion of PED-positive farms (30.89%) (Mai et al., unpublished). However, since its first detection, no specific PEDV control measures have been implemented by veterinary services to control the disease. Although vaccination or the feedback method has been applied on some pig farms, PEDV still occurs and frequently recurs. Our hypothesis is that the farm location and poor biosecurity measures for fomites, animals, and humans are the main risk factors for the nationwide transmission of PEDV in endemic areas. Some risk factors related to social and cultural practices in the Vietnamese swine industry could also play important roles in PEDV transmission among the pig population in Vietnam. Therefore, the objective of this study was to evaluate the potential risk factors for the widespread dissemination of PEDV in an endemic area with a case–control study. Theoutcome should extend our understanding of the dynamics of PEDV spread, in an effort to eliminate this economically important disease, which has emerged or re-emerged worldwide.

## Materials and Methods

### Data Collection

**Figure 1:**
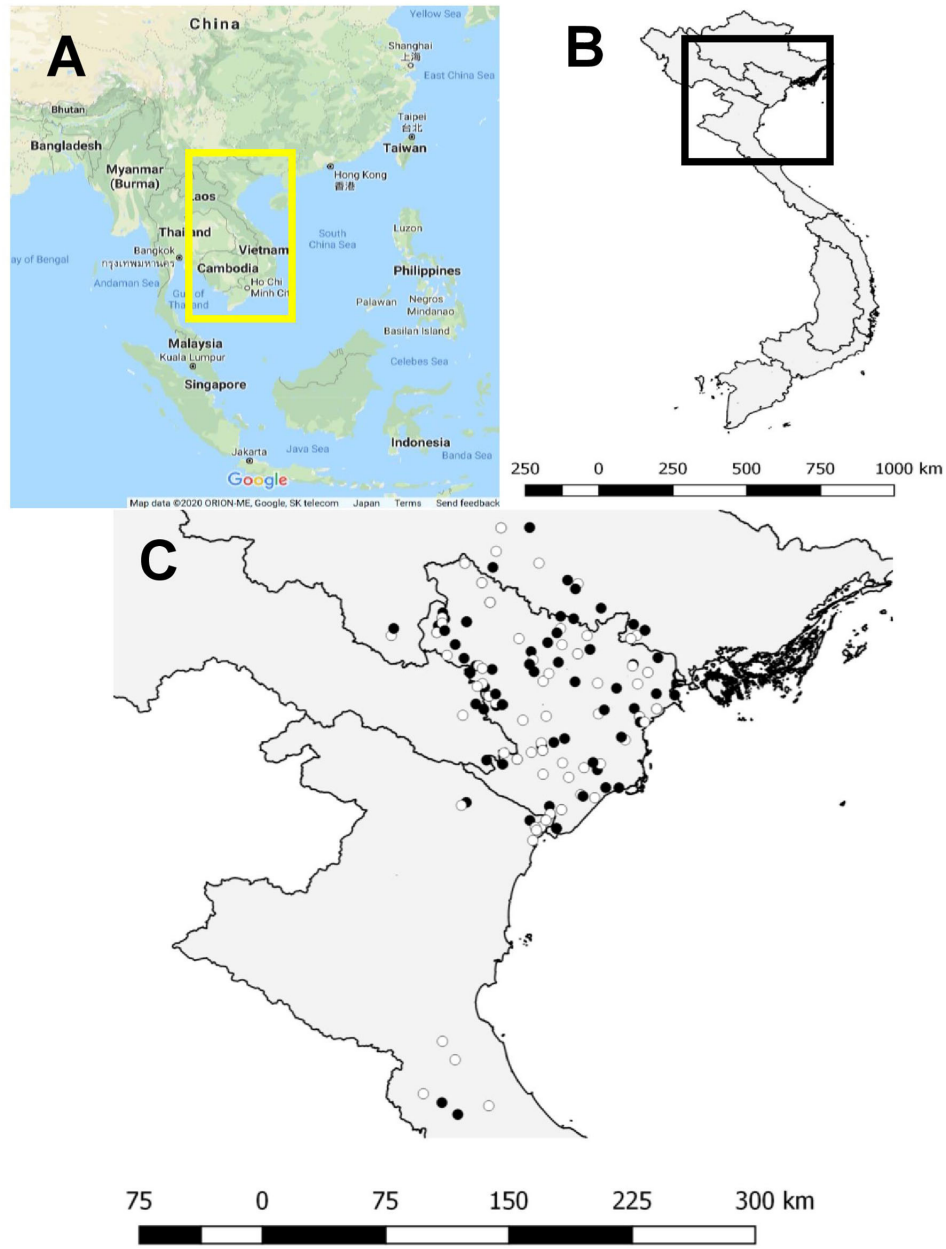
Map of the study area. **(A)** Map is from Google Maps (Map data©2020 Google) and the yellow rectangle shows Vietnam. **(B)** Map of Vietnam; black square indicates the study area. **(C)** Locations of the 187 pig farms in northern Vietnam involved in this study. Black dots indicate PEDV case farms and white dots indicate PEDV control farms. Geographical locations of the farms were mapped with the Quantum Geographic Information System.

In northern Vietnam, most piglets are produced and then transported to southernparts of the country. We have performed a survey between January 2018 and February 2019 to evaluate the proportion of PEDV-positive farms in a high-density pig population in northern Vietnam (Figure 1; the map was edited with PowerPoint from a screenshot of Google Maps [Map data©2020 Google]). The geographic location of Vietnam and the study area were mapped with the free, open-source Quantum Geographic Information System (QGIS) version 2.14.14 (https://www.qgis.org/en/site/). The required fecal sample size was estimated as 20 samples per farm. Therefore, on each farm, up to 20 fecal samples were collected from pigs in all ages. Then, 20 individual fecal samples from each farm were combined into two pooled samples (with a pooled size of 10 samples) for the test. Pooled samples were tested with reverse transcription (RT)–loop-mediated isothermal amplification (LAMP) (27). A “PEDV-positive farm” was defined as any swine herd with at least one positive result in two pooled samples from 20 samples collected from all-aged pigs. In total, 6,601 fecal samples were collected from 327 pig farms in northern Vietnam. The proportion of PEDV-positive farms was 30.9% (101 farms) and PEDV-positive farms were distributed throughout the study area. From the results of a PEDV survey of the pig population in northern Vietnam, there were 101 PEDV positive farms. All 101 PEDV-positive farms were selected as case farms, and 101 PEDV-negative farms, which were individually matched to a case farm based on the province in which they were located, were selected as the control farms for this study. The case and control farms were confirmed in 2018 with a PEDV survey to ensure that their management practices had not changed, even on the case farms, since 2018. We conducted a matched case–control study of the herd management practices for a period of 1 year (January—December, 2018). Questionnaires were sent to veterinarians or managers of these farms in July 2019, and the author (MTN) received the responses through the mail by the middle of August 2019.

### Questionnaire

The questionnaire was developed based on known or published risk factors for PED and other infectious diseases of pigs (6, 9, 11, 13, 28). The designed questionnaire was checked by the authors (SS and YS) and then pre-tested with a selected number of veterinarians for the clarity and appropriateness of its content, questions, and responses in terms of the local situation, before the start of the study. Modifications were made if necessary.

Using a postal questionnaire survey, a case–control study was conducted on the PEDV-positive and -negative farms in northern Vietnam. The final version of the questionnaire contained 49 questions focusing on four main categories (Table S1).

Based on the actual data in Vietnam, PED frequently recurred in pig farms that had the presence of PEDV outbreaks in the past. To know whether there is a risk of the persistence of PEDV in these farms or not, only two questions applied specifically to the case farms. The first question concerned whether there had been an occurrence of PEDV in the preceding 2 years. If the answer was “yes,” they were asked the second question, regarding how they dealt with PEDV on their farms.

### Statistical Analysis

The outcome of interest for this study was the binary response variable “PEDV status,” which took the value 1 if the farm was PEDV positive and 0 if the farm was PED negative according to RT–LAMP. The data captured with the questionnaire were entered into Microsoft Excel and all statistical analyses were performed with the statistical software R version 3.4.3 (CRAN, 2017). A univariate logistic regression analysis was performed to assess the association between PEDV status and each independent variable in the questionnaire. The significance of each explanatory variable was tested with the Wald test. Any variables significantly associated with PEDV status at the *P* <0.20 level were subsequently selected for multivariable modeling. Backwards stepwise explanatory variable selection, beginning with the least significant variable, was performed while observing the changes in Akaike's information criterion (AIC). The final model was obtained with *P* levels for the remaining variables of <0.05 and minimum AIC. After the main effects were identified, all possible two-way interactions were also examined. In this study, the total number of pigs did not correlate with PEDV status in the regression analysis. Therefore, to distinguish the influence of location (province) and the total number of pigs on the risk of PEDV, both variables were included as random effects in the multivariate model, by fitting generalized linear mixed-effects models. We used the lme4 package in R to analyze the fitted generalized linear mixed-effects models. The strengths of association between all the variables and the outcome were quantified by evaluating the odds ratios (ORs) and the corresponding 95% confidence intervals (CIs). The contribution of each variable in the final model of the risk of PEDV infection was quantified as the population attributable fraction (29). To avoid model convergence, multicollinearity was also tested with the variance inflation factor of explanatory variables (30).

### Ethical Considerations

Informed consent was obtained in written form (by signature) or orally from all participants, who agreed to participate after they had received written information about the study. Ethical approval for the study was granted by the Hanoi School of Public Health Institutional Review Board, Hanoi, Vietnam (approval number 019-405/DD-YTCC) and the Animal Ethics Committee of the University of Miyazaki's Faculty of Agriculture, Miyazaki, Japan (approval number 2017–541).

## Results

### Descriptive Statistics and Univariate Associations

In total, 187/202 (92.6%) respondents from 92/101 (91.1%) case farms and 95/101 (94.1%) control farms, located in a high-density pig-farming area in northern Vietnam, were included in this study (Figure 1). Some questionnaires had missing responses, which were excluded from the analysis. These farms were identified in a previous PEDV survey of the pig population in northern Vietnam. The data collected from these 187 farms were included in the analysis. In general, the number of pigs on the case farms was lower than the number on the control farms (mean numbers of pigs on the case and control farms were 1090 ± 1055.4 and 1533 ± 1661.4, respectively, *P* > 0.05). On average, the case farms had fewer vehicles visiting the farm per month than the control farms during the time of the study (mean numbers of vehicles per month on the case and control farms were 8.0 ± 4.3 and 8.4 ± 4.7, respectively, *P* > 0.05). However, the case farms contained more animal species other than pigs than the control farms (mean numbers of different animal species on the case and control farms were 3.6 ± 1.4 and 2.5 ± 1.5 species, respectively, *P* <0.05). Of the 92 case farms, 52.17% (48/92) had experienced PEDV in the preceding 2 years and these farms used feedback to control the PEDV outbreaks. Of these farms, 27.08% (13/48) reported that the PEDV-infected pigs on their farms were still moved to other farms or sold to an abattoir. Only 5.88% (11/187) of the farms in the study used a PEDV vaccine to prevent infection.

In total, there were 49 variables in the case-control study. The univariate associations of 49 variables that were considered possible risk factors for PEDV infection are presented in Table 1. Of these 9 variables of interest, 29 were associated with PEDV status (*P* <0.20).

**Table 1:** Results of a univariate analysis of location variables in the risk of *Porcine epidemic diarrhea virus* infection in a case–control study of northern Vietnamese pig farms in 2018.

**Groups**	**Variables**	**Category**	**No. of cases**	**Proportion of response (%)**	**No. of controls**	**Proportion of response (%)**	**OR (95% CI)**	***P*-value**
Farm location (*n* = 8)	Distance from farm to the closest farm	<200 m	24	26.1	17	17.9	1.96 (0.82–4.67)	0.127
		201–500 m	16	17.4	17	17.9	1.31 (0.52–3.26)	0.565
		501–1,000 m	34	37.0	36	37.9	1.31 (0.61–2.82)	0.487
		>1,000 m	18	19.6	25	26.3	Ref
	Distance from farm to the main road	<200 m	24	26.1	14	14.7	3.31 (1.34–8.21)	0.009
		201–500 m	26	28.3	15	15.8	3.35 (1.38–8.16)	0.007
		501–1,000 m	27	29.3	37	38.9	1.41 (0.64–3.13)	0.396
		>1,000 m	15	16.3	29	30.5	Ref
	Distance from farm to the residential area	<200 m	19	20.7	9	9.5	4.10 (1.53–10.98)	0.004
		201–500 m	24	26.1	25	26.3	1.86 (0.83–4.19)	0.13
		501–1,000 m	32	34.8	28	29.5	2.22 (1.02–4.81)	0.042
		>1,000 m	17	18.5	33	34.7	Ref
	Distance from farm to the irrigation system	<200	54	58.7	40	42.1	2.16 (1.08–4.32)	0.028
		201–500 m	18	19.6	23	24.2	1.25 (0.54–2.88)	0.596
		>500 m	20	21.7	32	33.7	Ref
	Distance from farm to the slaughterhouse	< =1,000 m	22	23.9	4	4.2	5.35 (1.68–17.07)	0.002
		Unknown	33	35.9	55	57.9	0.58 (0.31–1.10)	0.093
		>1,000 m	37	40.2	36	37.9	Ref
	Distance from farm to local market	<500 m	12	13.0	2	2.1	9.27 (1.73–49.66)	0.004
		500–1,000 m	26	28.3	21	22.1	1.91 (0.74–4.96)	0.179
		1,001–5,000 m	43	46.7	55	57.9	1.21 (0.51–2.85)	0.665
		>5,000 m	11	12.0	17	17.9	Ref
	Distance from barn to living room	<10 m	21	22.8	18	18.9	1.71 (0.69–4.25)	0.246
		10–20 m	21	22.8	23	24.2	1.34 (0.55–3.24)	0.517
		21–50 m	35	38.0	32	33.7	1.60 (0.71–3.62)	0.253
		>50 m	15	16.3	22	23.2	Ref
	Distance from barn to the pig loading/unloading place	< =50 m	62	67.4	49	51.6	1.94 (1.07–3.51)	0.028
		>50 m	30	32.6	46	48.4	Ref
Farm management (*n* = 20)	Farm status	Private	28	30.4	29	30.5	1.00 (0.53–1.86)	0.989
		Company	64	69.6	66	69.5	Ref
	Production type	FF	20	21.7	26	27.4	0.99 (0.49–1.98)	0.97
		FW	26	28.3	10	10.5	3.33 (1.46–7.61)	0.003
		WF	46	50.0	59	62.1	Ref
	Total pigs	<500	28	30.4	28	29.5	Ref
		500–1,000	31	33.7	24	25.3	1.29 (0.61–2.73)	0.502
		1,000–1,500	18	19.6	15	15.8	1.20 (0.51–2.84)	0.679
		>1,500	15	16.3	28	29.5	0.54 (0.24–1.21)	0.133
	All-in/all-out policy in each barn	No	27	29.3	23	24.2	1.30 (0.68–2.49)	0.427
		Yes	65	70.7	72	75.8		
	Pig movement	Pig addition	10	37.0	4	17.4	Ref
		Pig removal	9	33.3	11	47.8	0.33 (0.08–1.40)	0.127
		Both	8	29.6	4	17.4	0.8 (0.15–4.24)	0.793
	Separate place for pig movement	No	21	22.8	17	17.9	1.36 (0.66–2.78)	0.402
		Yes	71	77.2	78	82.1		
	Pig movement place is located on farm's property	No	17	18.5	15	15.8	1.21 (0.56–2.59)	0.625
		Yes	75	81.5	80	84.2		
	Truck through the same route at entrance and exit	Yes	89	96.7	87	91.6	2.73 (0.7–10.62)	0.134
		No	3	3.3	8	8.4		
	Source of trucks for the pig transport to the slaughterhouse	Slaughterhouse trucks	48	52.2	38	40.0		0.095
		Business operator trucks	44	47.8	57	60.0	1.64 (0.92–2.92)	
	Having a separate worker in isolation barn	No	64	69.6	57	60.0	1.52 (0.83–2.79)	0.171
		Yes	28	30.4	38	40.0		
	Opened barn type	Yes	18	19.6	14	14.7	1.41 (0.65–3.03)	0.381
		No	74	80.4	81	85.3		
	Water source	Direct	49	53.3	25	26.3	3.19 (1.73–5.89)	<0.001
		Indirect	43	46.7	70	73.7	Ref
	Feeding swill to pigs	Yes	4	4.3	0	0.0	Inf	0.04
		No	88	95.7	95	100.0		
	Having workers in farm	Yes	76	82.6	71	74.7	1.61 (0.79–3.27)	0.189
		No	16	17.4	24	25.3		
	Changing workers	Monthly	6	6.5	2	2.1	1.98 (0.37–10.44)	0.414
		6 months	12	13.0	7	7.4	1.13 (0.40–3.19)	0.816
		Yearly	17	18.5	33	34.7	0.34 (0.16–0.71)	0.004
		No	47	51.1	31	32.6	Ref
	Living place of workers after finishing work on the farm	Staying at farms	46	50.0	45	47.4	Ref
		Go home	8	8.7	5	5.3	1.13 (0.40–3.19)	0.458
		Both	32	34.8	38	40.0	0.34 (0.16–0.71)	0.543
	Waste treatment applies in your farm	No	9	9.8	3	3.2	3.33 (0.87–12.70)	0.065
		Yes	83	90.2	92	96.8		
	Manure application	Feed for fish	7	7.6	3	3.2	7.58 (1.75–32.78)	0.003
		Applied on land inside farm	25	27.2	17	17.9	4.78 (2.08–10.99)	<0.001
		Mixed type	44	47.8	23	24.2	6.22 (2.93–13.21)	<0.001
		Sold	16	17.4	52	54.7	Ref
	Share boars with other farms	Yes	10	10.9	11	11.6	0.93 (0.38–2.31)	0.878
		No	82	89.1	84	88.4		
	Addition ingredients in feed	Antibiotic	36	39.1	49	51.6	0.69 (0.29–1.60)	0.381
		Probiotic	4	4.3	4	4.2	0.93 (0.20–4.47)	0.931
		Both	37	40.2	28	29.5	1.23 (0.51–2.97)	0.64
		None	15	16.3	14	14.7	Ref
Biosecurity practice and health management (*n* = 11)	Disinfection of environment on premises	Monthly	4	4.3	4	4.2	1.03 (0.25–4.26)	0.963
		Weekly	88	95.7	91	95.8		
	Biosecurity practices apply to people inside farm	High	67	72.8	69	72.6	Ref
		Intermediate	23	25.0	21	22.1	1.13 (0.57–2.23)	0.729
		Low	2	2.2	5	5.3	0.41 (0.08–2.20)	0.285
	Biosecurity practices apply to visitors	High	55	59.8	63	66.3	Ref
		Intermediate	21	22.8	17	17.9	1.41 (0.68–2.95)	0.353
		Low	16	17.4	15	15.8	1.22 (0.55–2.70)	0.62
	Biosecurity practices apply at pig loading/unloading place	High	54	58.7	50	52.6	Ref
		Intermediate	26	28.3	22	23.2	1.09 (0.55–2.17)	0.797
		Low	12	13.0	23	24.2	0.48 (0.22–1.07)	0.071
	Time that vehicles must wait after disinfection to get into the farm	≤ 2 h	71	77.2	82	86.3	0.54 (0.25–1.15)	0.105
		>2 h	21	22.8	13	13.7	Ref
	Time that vehicles must wait after disinfection to get into the farm	<30 min	15	16.3	20	21.1	Ref
		30–60 min	55	59.8	46	48.4	1.59 (0.73–3.46)	0.237
		>60 min	22	23.9	29	30.5	1.01 (0.42–2.41)	0.979
	Diseases happen in farm	High	40	43.5	33	34.7	1.73 (0.59–5.05)	0.311
		Intermediate	45	48.9	52	54.7	1.24 (0.43–3.52)	0.69
		Low	7	7.6	10	10.5	Ref
	Vaccination applying in farm	High	42	45.7	47	49.5	Ref
		Intermediate	39	42.4	43	45.3	1.01 (0.56–1.85)	0.961
		Low	11	12.0	5	5.3	2.46 (0.79–7.67)	0.112
	Source of human food	Local market	78	84.8	72	75.8	1.69 (0.69–4.13)	0.25
		Supermarket	5	5.4	9	9.5	0.86 (0.22–3.43)	0.835
		Inside farm	9	9.8	14	14.7	Ref
	Human food including pig products	Yes	21	22.8	16	16.8	1.46 (0.71–3.02)	0.304
		No	71	77.2	79	83.2		
	Cook human food before entering farm	No	81	88.0	80	84.2	1.38 (0.60–3.19)	0.449
		Yes	11	12.0	15	15.8		
People, animal and vehicle contact (*n* = 10)	Visiting of vet	Daily	6	6.5	5	5.3	1.37 (0.29–6.53)	0.691
		Weekly	29	31.5	28	29.5	1.18 (0.38–3.70)	0.772
		Monthly	50	54.3	54	56.8	1.06 (0.36–3.13)	0.919
		No	7	7.6	8	8.4	Ref
	Other visitors	Yes	47	51.1	37	38.9	1.64 (0.92–2.93)	0.095
		No	45	48.9	58	61.1		
	Presence of wild birds inside farm	Yes	47	51.1	32	33.7	2.06 (1.14–3.71)	0.016
		No	45	48.9	63	66.3		
	Presence of rodents inside farm	Yes	86	93.5	59	62.1	8.75 (3.57–22.07)	<0.001
		No	6	6.5	36	37.9		
	Presence of chicken in farm	Yes	58	63.0	32	33.7	3.36 (1.84–6.12)	<0.001
		No	34	37.0	63	66.3		
	Presence of ducks in farm	Yes	31	33.7	23	24.2	1.59 (0.84–3.01)	0.152
		No	61	66.3	72	75.8		
	Presence of dog in farm	Yes	78	84.8	80	84.2	1.04 (0.47–2.31)	0.914
		No	14	15.2	15	15.8		
	Presence of cat in farm	Yes	31	33.7	14	14.7	2.94 (1.44–6.0)	0.002
		No	61	66.3	81	85.3		
	Vehicles visit another farm on the same day/trip	Yes	7	7.6	8	8.4	1.01 (0.34–2.97)	0.986
		Unknown	33	35.9	27	28.4	1.41 (0.75–2.65)	0.284
		No	52	56.5	60	63.2	Ref
	Number of truck vehicles visit to farm/month	High	14	15.2	22	23.2	0.58 (0.27–1.28)	0.177
		Intermediate	29	31.5	28	29.5	0.95 (0.49–1.84)	0.882
		Low	49	53.3	45	47.4	Ref

### Multivariable Associations

Because many variables (29 variables) were eligible for inclusion in the multivariable model, the selected variables were separated and categorized into four groups based on 4 main categories in Table S1 and then four corresponding models were established. Backward stepwise selection was performed until the *P-*values of all the remaining variables were <0.1 in the four corresponding models. A new model was established using the remaining variables in the four models (*P* <0.1) (6). Further stepwise simplification of the model has applied until the estimated regression coefficients for all the explanatory variables retained in the final model were significant on the Wald test at a *P* <0.05. Table 2 shows the results of the final model. Only three of the 29 variables in these four models remained significant risk factors for PEDV infection in the final model: farrow-to-wean production (OR = 3.35, 95% CI: 1.51–7.43), close proximity of the farm to the slaughterhouse (OR = 7.15, 95% CI: 2.36–21.70), and the presence of chickens on the farm (OR = 3.36, 95% CI: 1.84–6.12) (AIC = 124.2).

**Table 2:** Results of the final multivariate model of risk factors associated with *Porcine epidemic diarrhea virus* in a case–control study on northern Vietnamese pig farms in 2018.

**Variables**	**OR**	**95% CI**	**Coefficient**	**SE**	**Z-Statistic**	***P*-value**
Intercept			−0.9694	0.4587	−2.113	0.0346
Production type (FW)	3.35	1.51–7.43	1.6194	0.6473	2.502	0.0124
Near distance to the slaughterhouse (<1,000 m)	7.15	2.36–21.70	1.9391	0.7085	2.737	0.0062
Presence of chicken	3.36	1.84–6.12	1.1282	0.5646	1.998	0.0457

## Discussion

To the best of our knowledge, this is the first study to quantify the risk factors for the spread of PEDV in an endemic area using a case–control strategy based on a questionnaire survey. Only three of the 49 variables tested remained significant risk factors for PEDV spread in the final model. The three main risk factors for the spread of PEDV in an endemic area in Vietnam are the farrow-to-wean production type, close proximity of the farm to the slaughterhouse, and the presence of chickens on the farm. These factors were significantly associated with the PEDV status of the farms.

In this study, there was a strong relationship between the distance from the farm to the slaughterhouse and the PEDV status of the farm. Close proximity to the slaughterhouse (<1,000 m) increased the risk of PEDV infection 7.15-fold relative to that on farms further from the slaughterhouse. Population attributed fraction is the proportion of disease in the population that is due to expose (29, 31). The population attributable fraction for this risk factor was 20.57% (95% CI 10.26–29.70), so eliminating or completely preventing its effects would reduce the incidence of PEDV by 20.57%. Cross-contamination of farm vehicles is reported to occur at slaughterhouses (11, 13). There are few central slaughterhouses in Vietnam, and pigs are commonly slaughtered at a slaughtering point near the house of a butcher or pig trader to supply meat to traditional markets. This partly explains why 47.06% (88/187) of the farms in this study reported “unknown distance” in response to the question on this variable. In this study, 27.08% (13/48) of the farms that had experienced PEDV in the preceding 2 years still moved or sold infected pigs to slaughterhouses or other farms. Moving the infected pigs to slaughterhouses could be a risk for environmental contamination of facility because of pigs shedding the virus in feces which can be tracked back to farms. Moving infected pigs has been shown to play a role in the spread of PEDV among farms (13, 14). The open transportation of pigs and pig products from the slaughterhouse to the local market on motorbikes, which is a popular transport method in Vietnam, could also explain why variables related to the farm location, such as the distance from the farm to the main road (<500 m), the distance from the farm to the local market (<500 m), and the distance from the farm to the residential area (<200 m), were associated with the risk of PEDV infection in the univariate analysis. PEDV is known to persist on pig transportation vehicles especially if not disinfected post-use for transportation (13). All the reasons cited above could increase the risk of PEDV infection on farms close to slaughterhouses through the cross-contamination between animals, vehicles, fomites, and humans, and through the movement of animals or aerosol transmission. Increases in the risk of PEDV transmission have also been attributed to the movement of animals and aerosol transmission in previous studies (14, 32).

Another finding of the present study was the strong relationship between the presence of chickens on the farms and PEDV status (OR = 3.36, 95% CI: 1.84–6.12). Other animal species were also more frequently present on the case farms than on the control farms (average numbers of other animal species on the case and control farms were 3.6 ± 1.4 and 2.5 ± 1.5, respectively). Movements of animals between farms and other neighborhood features were indicated to be the most important factors associated with PEDV occurrence (33). Previous studies have also demonstrated that other animals on pig farms can transmit PEDV. PEDV was found in the tonsils in 4.2% of cats, suggesting that cats may play a role in the transmission of PEDV on swine farms (28). Experience in the USA suggests that the transmission of PEDV is related to bird traffic (32). Therefore, the transmission of PEDV on the case farms may be attributable to animal contact because animal species other than pigs were recorded on site. For example, 22.2% of the case farms had both cats and dogs, and another 22.2% of case farms had only cats (8). In our study, although the presence of other animal species (including wild birds, rodents, and cats) did not remain a risk factor in the final model, it was significantly associated with PEDV status in the univariate analysis. Furthermore, 31 of 92 (33.7%) case farms reported having ducks and 78/92 (84.78%) reported having dogs. Therefore, the presence of other animal species in general and especially the presence of chickens on the farms could play a role in the transmission of PEDV through animal contact and movement. Animal movement is an important mechanisms of pathogen transmission.

In this study, the farrow-to-wean production type was related to PEDV status, and had a 3.35-fold (95% CI: 1.51–7.43) higher risk of PEDV infection than other production systems. When PEDV infection occurs on a pig farm, it usually spreads among pigs of different ages. However, pigs display age-dependent resistance to pathogenic PEDV infection (34, 35). The virus accumulates and infects pregnant sows, and the subclinically infected sows transmit PEDV to the suckling piglets. The virus is then transferred to pigs of different ages (36). In another study, PEDV was considered to have been introduced in feeder pigs, fattening pigs, and adult pigs, and then spread to piglets (37). In the early phase of PEDV outbreaks, sow farms have had the highest incidence of PEDV (80.0%) (19, 32). However, after an acute outbreak, PEDV may remain in the farrowing unit because of poor biosecurity or persist in pigs in weaning or growing–finishing units, where the virus circulates (32). The number of sows on a farm is thought to play a role in the persistence of PEDV after the original outbreak (37). Of the case farms in the present study, 52.17% (48/92) had experienced PEDV in the preceding 2 years, and 68.75% (33/48) of these were sow farms (farrow-to-finish and farrow to wean production types). These outbreaks could have been caused by PEDV that was still circulating on these farms after the previous outbreak, which acted as the source of the recurrent epidemic outbreaks. This may explain why PED outbreaks occur periodically in endemic regions.

The application of manure as a biosecurity issue did not remain a risk factor for PEDV spread in the final model. However, there was a strong relationship between manure application and PEDV status in the univariate analysis. A previous study indicated that PEDV may persist in a herd long after its clinical impact (32). Previous studies have also provided evidence that PEDV can survive for up to 28 days in manure between −20 and 4 °C, and for up to 9 months in infected earthen manure stored at temperatures ranging from −30 to 23 °C (32, 38). In the present study, 68/187 farms sold manure from the farm, which could be a risk factor for the wide transmission of PEDV to other farms if the virus was present in the manure. Risk factors based on social or cultural practices, such as water sources (direct from drilled wells or irrigation systems), were not included in the final model, but were associated with PEDV status in the univariate analysis. In Vietnam, water for pig rearing can be taken from the irrigation system surrounding the farm, drilled wells, or fish ponds. Dead pigs are often thrown into the river. Therefore, there is a high risk of introducing PEDV into the irrigation system or groundwater, which could then be dispersed by the water flow. Therefore, to prevent further transmission of PEDV, it is necessary to raise public awareness about the risk entailed by social and cultural practices in pig raising in Vietnam.

Our study had several limitations. The accuracy of the information depended partly on the professional ability of each veterinarian or farm manager, whose knowledge of the epidemiology of PEDV could be limited. Second, bias may have been introduced by the time lag between the PEDV occurrence on farms and the questionnaire survey. Third, the respondents may have answered questions involving sensitive issues incorrectly. Typical examples are how sick pigs (selling) or dead pigs (throw away) are dealt with. In addition, the selection of PEDV-negative farm could affect the analysis for the identification of risk factors.

## Conclusions

This is the first study to identify the three main risk factors for the spread of PEDV in an endemic area: the presence of chickens on the farm, close proximity to the slaughterhouse, and the farrow-to-wean production type. It is also the first study to show the distance to the slaughterhouse can play an important role in transmitting PEDV and to indicate this was the principal risk factor associated with the endemic area. In addition, the mechanical transmission by the presence of chicken in the farm that caused by the movement of chicken could be some way to explain the widespread of PEDV.

## Data Availability Statement

All datasets presented in this study are included in the article/Supplementary Material.

## Ethics Statement

The studies involving human participants were reviewed and approved by Informed consent was obtained in written form (by signature) or orally from all participants, who agreed to participate after they had received written information about the study. Ethical approval for the study was granted by the Hanoi School of Public Health Institutional Review Board, Hanoi, Vietnam (Approval Number 019-405/DD-YTCC) and the Animal Ethics Committee of the University of Miyazaki's Faculty of Agriculture, Miyazaki, Japan (Approval Number 2017–541). The patients/participants provided their written informed consent to participate in this study.

## Author Contributions

TM designed the questionnaire, performed the study, analyzed the data, and drafted the manuscript. TB participated in the distribution and collection the questionnaires. YS participated in the design of the questionnaire, reviewed the drafts of the manuscript, and suggested revisions. TH, SM, HD, WF, and JN reviewed the drafts and suggested revisions. SS conceptualized and supervised the study, and analyzed and revised the manuscript. All authors read and approved the final manuscript.

## Conflict of Interest

TB is an employee of Branch of Cargill Vietnam Co., Ltd. The remaining authors declare that the research was conducted in the absence of any commercial or financial relationships that could be construed as a potential conflict of interest.

## References

[1] Sun R Cai RJ Chen YQ Liang PS Chen DK Song CX. Outbreak of porcine epidemic diarrhea in suckling piglets, China. Emerg Infect Dis. (2012) 18:161–3. 10.3201/eid1801.111259 22261231 PMC3381683

[2] Kochhar HS. Canada: porcine epidemic diarrhea in Canada: an emerging disease case study. Can Vet J. (2014) 55:1048–9. 25392546 PMC4204834

[3] Lee C. *Porcine epidemic diarrhea virus*: an emerging and re-emerging epizootic swine virus. Virol J. (2015) 12:193. 10.1186/s12985-015-0421-2 26689811 PMC4687282

[4] Niederwerder MC Hesse RA. Swine enteric coronavirus disease: a review of 4 years with *Porcine epidemic diarrhoea virus* and porcine deltacoronavirus in the United States and Canada. Transbound Emerg Dis. (2018) 65:660–75. 10.1111/tbed.12823 29392870 PMC7169865

[5] Lin CN Chung WB Chang SW Wen CC Liu H Chien CH. US-like strain of *Porcine epidemic diarrhea virus* outbreaks in Taiwan, 2013-2014. J Vet Med Sci. (2014) 76:1297–99. 10.1292/jvms.14-0098 24898162 PMC4197162

[6] Toyomaki H Sekiguchi S Sasaki Y Sueyoshi M Makita K. Factors associated with farm-level infection of porcine epidemic diarrhea during the early phase of the epidemic in Japan in 2013 and 2014. Prev Vet Med. (2018) 150:77–85. 10.1016/j.prevetmed.2017.12.008 29406087

[7] Kim SH Lee JM Jung J Kim IJ Hyun BH Kim HI. Genetic characterization of *Porcine epidemic diarrhea virus* in Korea from 1998 to 2013. Arch Virol. (2015) 160:1055–64. 10.1007/s00705-015-2353-y 25666198 PMC7086719

[8] Perri AM Poljak Z Dewey C Harding JCS O'Sullivan TL. An epidemiological investigation of the early phase of the porcine epidemic diarrhea (PED) outbreak in Canadian swine herds in 2014: a case-control study. Prev Vet Med. (2018) 150:101–9. 10.1016/j.prevetmed.2017.12.009 29406076

[9] Pasick J Berhane Y Ojkic D Maxie G Embury-Hyatt C Swekla K. Investigation into the role of potentially contaminated feed as a source of the first-detected outbreaks of porcine epidemic diarrhea in Canada. Transbound Emerg Dis. (2014) 61:397–410. 10.1111/tbed.12269 25098383 PMC4282400

[10] Kim Y Yang M Goyal SM Cheeran MCJ Torremorell M. Evaluation of biosecurity measures to prevent indirect transmission of *Porcine epidemic diarrhea virus*. BMC Vet Res. (2017) 13:89. 10.1186/s12917-017-1017-4 28381304 PMC5382501

[11] Sasaki Y Alvarez J Sekiguchi S Sueyoshi M Otake S Perez A. Epidemiological factors associated to spread of porcine epidemic diarrhea in Japan. Prev Vet Med. (2016) 123:161–7. 10.1016/j.prevetmed.2015.11.002 26588869

[12] Sasaki Y Toyomaki H Sekiguchi S Sueyoshi M Makita K Otake S. Spatial dynamics of porcine epidemic diarrhea (PED) spread in the southern Kyushu, Japan. Prev Vet Med. (2017) 144:81–8. 10.1016/j.prevetmed.2017.05.025 28716208

[13] Lowe J Gauger P Harmon K Zhang J Connor J Yeske P. Role of transportation in spread of *Porcine epidemic diarrhea virus* infection, United States. Emerg Infect Dis. (2014) 20:872–4. 10.3201/eid2005.131628 24750785 PMC4012813

[14] O'Dea EB Snelson H Bansal S. Using heterogeneity in the population structure of U.S. swine farms to compare transmission models for porcine epidemic diarrhoea. Sci Rep. (2016) 6:22248. 10.1038/srep22248 26947420 PMC4780089

[15] Boniotti MB Papetti A Bertasio C Giacomini E Lazzaro M Cerioli M. *Porcine epidemic diarrhoea virus* in Italy: disease spread and the role of transportation. Transbound Emerg Dis. (2018) 65:1935–42. 10.1111/tbed.12974 30094946 PMC7169760

[16] Dee S Neill C Singrey A Clement T Cochrane R Jones C. Modeling the transboundary risk of feed ingredients contaminated with *Porcine epidemic diarrhea virus*. BMC Vet Res. (2016) 12:51. 10.1186/s12917-016-0674-z 26968372 PMC4788872

[17] Bowman AS Krogwold RA Price T Davis M Moeller SJ. Investigating the introduction of *Porcine epidemic diarrhea virus* into an Ohio swine operation. BMC Vet Res. (2015) 11:38. 10.1186/s12917-015-0348-2 25881144 PMC4334577

[18] Dee S Clement T Schelkopf A Nerem J Knudsen D Christopher-Hennings J. An evaluation of contaminated complete feed as a vehicle for *Porcine epidemic diarrhea virus* infection of naive pigs following consumption via natural feeding behavior: proof of concept. BMC Vet Res. (2014) 10:176. 10.1186/s12917-014-0176-9 25091641 PMC4363994

[19] Alvarez J Goede D Morrison R Perez A. Spatial and temporal epidemiology of porcine epidemic diarrhea (PED) in the Midwest and Southeast regions of the United States. Prev Vet Med. (2016) 123:155–60. 10.1016/j.prevetmed.2015.11.003 26586344

[20] Alvarez J Sarradell J Morrison R Perez A. Impact of porcine epidemic diarrhea on performance of growing pigs. PLoS ONE. (2015) 10:e0120532. 10.1371/journal.pone.0120532 25768287 PMC4359118

[21] Do TD Nguyen TT Suphasawatt P Roongroje T Genetic characterization of *Porcine epidemic diarrhea virus* (PEDV) isolates from Southern Vietnam during 2009–2010 outbreaks. Thai J Vet Med. (2011) 41:55–64. 10.1002/vms3.256

[22] Vui DT Thanh TL Tung N Srijangwad A Tripipat T Chuanasa T. Complete genome characterization of *Porcine epidemic diarrhea virus* in Vietnam. Arch Virol. (2015) 160:1931–8. 10.1007/s00705-015-2463-6 26026958

[23] Vui DT Tung N Inui K Slater S Nilubol D. Complete genome sequence of *Porcine epidemic diarrhea virus* in Vietnam. Genome Announc. (2014) 2:e00753–14. 10.1128/genomeA.00753-14 25125639 PMC4132615

[24] Kim YK Lim SI Lim JA Cho IS Park EH Le VP. A novel strain of *Porcine epidemic diarrhea virus* in Vietnamese pigs. Arch Virol. (2015) 160:1573–7. 10.1007/s00705-015-2411-5 25864174

[25] Diep NV Sueyoshi M Izzati U Fuke N Teh APP Lan NT. Appearance of US-like *Porcine epidemic diarrhoea virus* (PEDV) strains before US outbreaks and genetic heterogeneity of PEDVs collected in Northern Vietnam during 2012–2015. Transbound Emerg Dis. (2018) 65:e83–93. 10.1111/tbed.12681 28758349 PMC7169849

[26] Choe SE Park KH Lim SI Le VP Hien NB Thach PN. Complete genome sequence of a *Porcine epidemic diarrhea virus* strain from Vietnam, HUA-14PED96, with a large genomic deletion. Genome Announc. (2016) 4:e00002–16. 10.1128/genomeA.00002-16 26893409 PMC4759056

[27] Mai T Nguyen VD Yamazaki W Okabayashi T Mitoma S Notsu K. Development of pooled testing system for porcine epidemic diarrhoea using real-time fluorescent reverse-transcription loop-mediated isothermal amplification assay. BMC Vet Res. (2018) 14:172. 10.1186/s12917-018-1498-9 29843733 PMC5975689

[28] Truong QL Seo TW Yoon BI Kim HC Han JH Hahn TW. Prevalence of swine viral and bacterial pathogens in rodents and stray cats captured around pig farms in Korea. J Vet Med Sci. (2013) 75:1647–50. 10.1292/jvms.12-0568 23892461 PMC3942947

[29] Whittemore AS. Statistical methods for estimating attributable risk from retrospective data. Stat Med. (1982) 1:229–43. 10.1002/sim.4780010305 7187096

[30] O'brien RM A Caution regarding rules of thumb for variance inflation factors. Qual Quant. (2007) 41:673–90. 10.1007/s11135-006-9018-6

[31] Brooks-Pollock E Danon L. Defining the population attributable fraction for infectious diseases. Int J Epidemiol. (2017) 46:976–82. 10.1093/ije/dyx055 28472445 PMC5837626

[32] Beam A Goede D Fox A McCool MJ Wall G Haley C. A *Porcine epidemic diarrhea virus* outbreak in one geographic region of the United States: descriptive epidemiology and investigation of the possibility of airborne virus spread. PLoS ONE. (2015) 10:e0144818. 10.1371/journal.pone.0144818 26709512 PMC4692406

[33] Machado G Vilalta C Recamonde-Mendoza M Corzo C Torremorell M Perez A. Identifying outbreaks of *Porcine epidemic diarrhea virus* through animal movements and spatial neighborhoods. Sci Rep. (2019) 9:457. 10.1038/s41598-018-36934-8 30679594 PMC6345879

[34] Shibata I Tsuda T Mori M Ono M Sueyoshi M Uruno K. Isolation of *Porcine epidemic diarrhea virus* in porcine cell cultures and experimental infection of pigs of different ages. Vet Microbiol. (2000) 72:173–82. 10.1016/S0378-1135(99)00199-6 10727829 PMC7117361

[35] Koike N Mai TN Shirai M Kubo M Hata K Marumoto N. Detection of neutralizing antibody against *Porcine epidemic diarrhea virus* in subclinically infected finishing pigs. J Vet Med Sci. (2018) 80:1782–6. 10.1292/jvms.18-0132 30282841 PMC6261828

[36] Wang D Fang L Xiao S. Porcine epidemic diarrhea in China. Virus Res. (2016) 226:7–13. 10.1016/j.virusres.2016.05.026 27261169 PMC7114554

[37] Pensaert MB Martelli P. Porcine epidemic diarrhea: a retrospect from Europe and matters of debate. Virus Res. (2016) 226:1–6. 10.1016/j.virusres.2016.05.030 27317168 PMC7132433

[38] Tun HM Cai Z Khafipour E. Monitoring survivability and infectivity of *Porcine epidemic diarrhea virus* (PEDv) in the infected on-farm earthen manure storages (EMS). Front Microbiol. (2016) 7:265. 10.3389/fmicb.2016.00265 27014197 PMC4783413

