# Supplementary material for: Evaluating the Risk Factors for Porcine Epidemic Diarrhea Virus Infection in an Endemic Area of Vietnam
Source: Front Vet Sci. 2020 Jul 29;7:433. doi: 10.3389/fvets.2020.00433 (PMC7403480; doi:10.3389/fvets.2020.00433)
Supplement: Supplementary file 1 [file Table_1.DOCX]

**Table S1. Variables used to assess the risk factors in a case–control study of PEDV and their definitions**

| **Groups** | **Variables** | **Definition and category** |
| --- | --- | --- |
| Farm location  (n=8) | Distance to closest farm | <200m, 200-500m, 500-1000m, >1000m |
|  | Distance from the farm to the main roads | <200m, 200-500m, 500-1000m, >1000m |
|  | Distance from the farm to the river | <200m, 200-500m, >500m |
|  | Distance from the farm to a slaughterhouse | <=1000m, >1000m |
|  | Distance from the farm to a local market or livestock market | <500m, 500-1000m, 1000-5000m, >5000m |
|  | Distance from the farm to the residential area | <200m, 200-500m, 500-1000m, >1000m |
|  | Distance from pig movement place to the barn | <50m, >50m |
|  | Distance from the barn to living room of workers | <10m, 10 - 20m, 20 - 50m, >50m |
| Farm management  (n=20) | Total pigs | Total number of pigs in the farm (Continuous variable) |
|  | Production system | Farrow-to-finish, Farrow-to-wean and wean-to-finish |
|  | Farm status | Private/Company |
|  | All-in/all-out policy | Yes/No |
|  | Pig movement | Pig addition, Pig removal, Both |
|  | Separate place for pig movement | Yes/No |
|  | Pig movement place is located on farm's property | Yes/No |
|  | Truck through the same route at entrance and exit | Yes/No |
|  | Source of trucks for the pig transport to the slaughterhouse | Slaughterhouses' trucks, Business operator’s trucks |
|  | Opened barn type | Yes/No |
|  | Water source usage for pig raising | Direct (from drilled well or irrigation system), Indirect (from drilled well with chlorine in tank) |
|  | Feeding swill to pigs | Yes/No |
|  | Having workers in farm | Yes/No |
|  | Changing workers | Monthly, six months, yearly, No |
|  | Isolation barn | Yes/No |
|  | Living place of workers after working | Staying at farms, Go home, Mixed |
|  | Waste treatment in the farm | Yes/No |
|  | Manure application | Fish feeding, applying on land inside farm, Sold, Mixed type |
|  | Share boars with other farms | Yes/No |
|  | Addition ingredients in feed | Antibiotic, Probiotic, Both, None |
| Biosecurity practice and health management  (n=11) | Disinfection of environment on premises | Weekly disinfection, Monthly disinfection |
|  | To people inside farm | High (Applied more than 5 items),  Intermediate (Applied 3 to 4 items),  Low (Applied less than 2 items)  The items were: Required to shower before operation, Change clothes and footwear before operation, Wear rubber boots, Doing disinfection in lime trays before and after each barn, required to shower after operation, regularly wash and disinfect clothes/boots |
|  | To visitors | High (Applied more than 6 items),  Intermediate (Applied 4 to 5 items),  Low (Applied less than 3 items)  The items were: Required to shower before operation, Change clothes and footwear before operation, Wear rubber boots, Doing disinfection in lime trays before and after each barn, Required to shower after operation, Regularly wash and disinfect clothes/boots, Must wait 24 h or more before visiting. |
|  | At pig loading/unloading: | High (Applied more than 7 items),  Intermediate (Applied 3 to 6 items),  Low (Applied less than 2 items)  The items were: Washing applied to the chute floor after each loading/unloading, Disinfection applied to the chute floor after each loading/unloading, The driver never step in an area at any time during or after the loading/unloading process, Wash truck before loading, Disinfect truck before loading, Wash and disinfection performed separately before loading, Wash trucks and trailers prior to visiting a site after leaving another swine site, Wash animal area of truck, Disinfect animal area of truck, Wash outside area of truck, Disinfect outside area of truck |
|  | Time that vehicles must wait after disinfection to get into the farm | < 2 h, 2-4 h, > 4h |
|  | Time for moving in/out pig/feed from vehicles | <30 min, 30 - 60 min, > 60 min |
|  | Health management |  |
|  | Disease status | High (Have more than 4 diseases),  Intermediate (Have 1 to 3 diseases),  Low (No disease)  The items were: Porcine reproductive and respiratory syndrome (PRRS), Pasteurellosis, Classical swine fever (CSF), Salmonella, Erysipelas, Mycoplasma,Transmissible gastroenteritis (TGE), Foot and Mouth disease (FMD), Porcine circovirus associated disease (PCVAD), Streptococcus suis, Atrophic rhinitis (AR), Glasser’s disease, Aujeszky’s disease (AD), Actinobacillus pleuropneumonia (APP), E. coli, Clostridium, Roundworm, Swine flu |
|  | Vaccine program | High (Applied more than 9 vaccines),  Intermediate (Applied 6 to 8 vaccines),  Low (Applied less than 5 vaccines)  The items were: PRRS, Pasteurellosis, CSF, Salmonella, Erysipelas, Mycoplasma, TGE, FMD, PCVAD, Streptococcus suis, Glasser’s disease, AD, APP, E. coli, Clostridium, Roundworm |
|  | Source of human food | Local market, Supermarket, Inside farm |
|  | Human food including pig products | Yes/No |
|  | Cook human food before entering farm | Yes/No |
| People, animal and vehicle contact  (n =10) | Visiting of vet | Daily, Weekly, Monthly, NO |
|  | Other visitors | Yes/No (Other visitors as neighbor, family members, pig traders or butchers, electric/gas company staff) |
|  | Animal contact |  |
|  | Presence of wild birds inside farm | Yes, No |
|  | Presence of rodents inside farm | Yes, No |
|  | Presence of chicken in farm | Yes, No |
|  | Presence of ducks in farm | Yes, No |
|  | Presence of dog in farm | Yes, No |
|  | Presence of cat in farm | Yes, No |
|  | Vehicles |  |
|  | Total trucks/month | Number of vehicles visit to farm per month (Continuous variable) |
|  | Vehicles visit another farm on the same day or same trip | Yes/No |
